# Interobserver agreement regarding the Fleischner Society diagnostic criteria for usual interstitial pneumonia patterns on computed tomography

**DOI:** 10.1590/0100-3984.2021.0033

**Published:** 2022

**Authors:** Stephanie Sander Westphalen, Felipe Soares Torres, Mateus Samuel Tonetto, Juliana Fischman Zampieri, Giovanni Brondani Torri, Tiago Severo Garcia

**Affiliations:** 1 Radiology Department, Hospital de Clínicas de Porto Alegre (HCPA), Porto Alegre, RS, Brazil.; 2 Radiology Department, Hospital Moinhos de Vento, Porto Alegre, RS, Brazil.; 3 Universidade Federal do Rio Grande do Sul (UFRGS), Porto Alegre, RS, Brazil.; 4 Department of Medical Imaging, University of Toronto, Toronto, ON, Canada.

**Keywords:** Lung diseases, interstitial/diagnosis, Tomography, X-ray computed/methods, Observer variation, Idiopathic pulmonary fibrosis/diagnostic imaging, Doenças pulmonares intersticiais/diagnóstico, Tomografia computadorizada/métodos, Variações dependentes do observador, Fibrose pulmonar idiopática/diagnóstico por imagem

## Abstract

**Objective:**

To assess interobserver agreement among radiologists regarding the current Fleischner Society diagnostic criteria for usual interstitial pneumonia (UIP) patterns on computed tomography (CT).

**Materials and Methods:**

Using the Fleischner Society criteria for UIP CT patterns, five raters, working independently, categorized the high-resolution CT (HRCT) scans of 44 patients with interstitial lung disease who underwent lung biopsy. The raters also evaluated the presence, extent, and distribution of the most relevant imaging findings, as well as indicating their level of confidence in the most likely diagnosis and in up to three diagnostic hypotheses.

**Results:**

There was moderate to substantial interobserver agreement regarding the UIP patterns on HRCT—kappa statistic (κ) = 0.59-0.61. Interobserver agreement for the binary scores was substantial (κ = 0.77-0.79), whereas that for the presence of honeycombing was almost perfect (κ = 0.81-0.96). There was agreement regarding at least one of the three diagnostic hypotheses in only 36.4% of the cases. For the level of confidence in the most likely diagnosis, there was only slight to fair agreement (κ = 0.19-0.21).

**Conclusion:**

Interobserver agreement regarding the current Fleischner Society CT criteria for UIP was moderate to substantial among raters with varying levels of experience. There was only slight to fair agreement regarding the diagnostic hypotheses and for the level of confidence in the most likely diagnosis.

## Abstract (pt)

**Objetivo:**

Avaliar a concordância interobservador entre radiologistas para os critérios atuais da Fleischner Society para categorias diagnósticas de pneumonia intersticial usual (PIU) em tomografia computadorizada (TC).

**Materiais e Métodos:**

Cinco observadores categorizaram independentemente as imagens de TC de 44 pacientes com doença pulmonar intersticial que foram submetidos a biópsia pulmonar empregando as últimas categorias de diagnóstico da Sociedade Fleischner para UIP. Também foram avaliadas presença, extensão e distribuição dos achados de imagem mais relevantes, bem como a confiança no diagnóstico mais provável e em até três hipóteses diagnósticas.

**Resultados:**

Houve concordância moderada a alta para as categorias diagnósticas entre os observadores (κ = 0,59-0,61). A concordância interobservador para a pontuação binária foi alta (κ = 0,77-0,79), enquanto para a presença de faveolamento foi considerada de alta a muito alta (κ = 0,81-0,96). Houve concordância em uma das três hipóteses diagnósticas em apenas 36,4% dos casos. Baixa concordância foi encontrada para o diagnóstico mais provável (κ = 0,19-0,21).

**Conclusão:**

A concordância entre observadores para os critérios atuais de TC da Fleischner Society para UIP foi moderada a alta entre observadores com diferentes níveis de experiência. Houve baixa concordância nas hipóteses diagnósticas e quanto ao grau de confiança no diagnóstico primário.

## INTRODUCTION

Interstitial lung diseases (ILDs) comprise a heterogeneous group of more than 200 rare inflammatory pathologies that usually present with diffuse pulmonary infiltrates. In many cases, it is not possible to identify the etiological factors^(1,2)^. The diagnosis is usually challenging and requires not only collaboration between different specialists but also the ability to interpret and communicate information that is often conflicting. Idiopathic pulmonary fibrosis (IPF) is the most common ILD and has the worst prognosis. However, drugs that can delay disease progression and loss of lung function have recently been approved and are also being considered in the treatment of other progressive fibrosing ILDs^(3,4)^.

In 2018, two major guidelines on IPF diagnostic criteria were published, both based on an extensive review of the available medical literature, including clinical, radiological, and pathological aspects^(5,6)^. High-resolution computed tomography (HRCT) of the chest is a central element in the investigation, being used for therapeutic decisions and to indicate additional diagnostic procedures. Those new guidelines reformulated the HRCT-based IPF/usual interstitial pneumonia (IPF/UIP) diagnostic categories, which aim to estimate the probability of HRCT findings corresponding to the UIP pattern in patients with clinically suspected IPF. Various studies have evaluated the diagnostic accuracy of HRCT in the context of diffuse lung diseases. Many of those studies have shown low diagnostic confidence, together with intraobserver and interobserver disagreement, as well as disagreement among clinicians, radiologists, and pathologists^(7-9)^. However, there are few objective data on interobserver agreement regarding the IPF/UIP diagnostic categories proposed for HRCT, especially in relation to new therapeutic approaches and guidelines.

The main objective of this study was to evaluate interobserver agreement between radiologists regarding the current IPF/UIP diagnostic categories based on the most recent classification proposed by the Fleischner Society, published in February 2018^(5)^.

## MATERIALS AND METHODS

This was an observational, cross-sectional, single-center study conducted at a tertiary care hospital. The study population consisted of 57 patients with suspected ILD who were selected by searching the hospital database for all codes of surgical procedures that could have been performed for the histological diagnosis of ILD (surgical lung biopsy, pulmonary segmentectomy, and pulmonary lobectomy). The database was searched for the period from January 2010 to February 2019, because it was expected that a sufficient number of patients would have undergone such procedures and been evaluated by HRCT during that period.

The inclusion criteria were having undergone surgical lung biopsy for suspected ILD between January 2010 and February 2019, having undergone chest HRCT at the same institution, and having a pathology report available in the medical records. Patients for whom the medical records were inaccurate were excluded, as were those for whom clinical or histological data were unavailable, those in whom the chest HRCT protocol was inappropriate/insufficient for diagnosis, and those in whom the final diagnosis was an infectious disease, focal disease, or other disease not within the scope of this study. The interval between lung biopsy and HRCT was not used as an exclusion criterion, because the main objective of this study was specifically to evaluate interobserver agreement regarding a HRCT-based classification, and that variable was therefore considered irrelevant. In addition, considering the rare nature of the studied diseases, losing a few patients could significantly impact the statistical power of the results.

We analyzed and compared the readings of four chest radiologists, with 4-20 years of experience, and a radiology resident at the end of their third year of training. The analysis of the HRCT images was objective and systematic, evaluating the presence and distribution of lesions and predominant changes. One to three diagnostic hypotheses were obtained; the first hypothesis considered by each radiologist was classified according to the subjective level of diagnostic confidence, from 1 to 3 (definite, probable, and possible). At the end, the evaluator categorized the set of findings according to UIP probability based on the current Fleischner Society consensus (Table 1) and recorded the information on a standardized data collection form. No clinical or histological information was provided to the radiologists, who had access only to the age and sex of the patient at the time of image acquisition. The raters were not trained and were informed only about the bibliography as a basis for categorizing the findings^(5)^. The data were entered into and stored in a database controlled by the lead investigator. As a means of control and comparison, the imaging findings were correlated with the clinical impression and with the pathology report of the lung biopsy.

**Table 1:** Diagnostic IPF/UIP categories on chest HRCT*.

Parameter	Typical UIP	Probable UIP	Indeterminate for UIP	non-IPF diagnosis
Distribution	Predominantly basal (occasionally diffuse) and subpleural; heterogeneous	Predominantly basal and subpleural; heterogeneous	Variable or diffuse	Fibrosis predominating in the middle or upper third of the lungs; peribronchovascular predominance sparing subpleural regions
Findings	Honeycombing; reticular pattern with peripheral traction bronchiectasis or bronchiolectasis^†^; no findings consistent with a non-IPF diagnosis	Reticular pattern with peripheral traction bronchiectasis or bronchiolectasis^†^; no honeycombing; no findings consistent with a non-IPF diagnosis	Signs of fibrosis associated with discrete or inconspicuous findings consistent with a nonIPF diagnosis	Any of the following: predominance of consolidation; extensive and insulated ground-glass opacity (with no acute exacerbation); mosaic attenuation and extensive air trapping; diffuse nodules or cysts

* Source: Lynch et al.^(5)^.

† Ground-glass opacity attenuation can be superimposed on the reticular pattern; usually related to fibrosis in these cases. However, isolated ground-glass opacity is not expected in IPF/UIP and suggests acute exacerbation or consistency with a non-IPF diagnosis when present.

### Statistical analysis

The IBM SPSS Statistics software package, version 22.0 (IBM Corp., Armonk, NY, USA) was used for the statistical analysis. We evaluated interobserver agreement regarding diagnostic categories and lesions by calculating the kappa (κ) statistic—in R, version 3.5.1, with the KappaM command in the DescTools package and kappam.fleiss command in the irr package—considering a standard error and 95% confidence interval. Interobserver agreement was categorized, on the basis of the κ value, as none to slight (κ ≤ 0.20), fair (κ = 0.21-0.40), moderate (κ = 0.41-0.60), substantial (κ = 0.61-0.80), or almost perfect (κ = 0.81-1.00).

Imaging findings, diagnostic hypotheses, and other data were subjected to descriptive analyses, and absolute and relative frequencies. Values of *p* < 0.05 were considered statistically significant.

### Sample size

The sample size was calculated on the basis of the expected rate of 95% correct interstitial pathology diagnoses desired by radiologists, with a real estimate of 80% (difference = 15%). The sample size calculation was based on the article authored by Scally et al.^(10)^. To achieve a power of 80% at a 5% level of significance, it was deemed necessary to include 46 patients with ILD.

### Ethical considerations

This study was conducted in accordance with the ethical standards established in Brazilian National Health Council Resolution no. 466/2012 and was approved by the local research ethics committee (Reference no. 82441517.1.0000.5327), respecting the bioethical principles of autonomy, beneficence, nonmaleficence, veracity, and confidentiality. The requirement for written informed consent was waived because of the retrospective nature of the study. All of the authors signed a confidentiality agreement to ensure the anonymity of the data obtained from the electronic medical records of the hospital.

## RESULTS

We identified 57 patients who had undergone surgical lung biopsy to diagnose diffuse lung disease between January 2010 and February 2019. Thirteen patients were excluded because HRCT images were unavailable or because the imaging findings or final diagnosis were not within the scope of the study (e.g., those of airway disease, respiratory infections, focal pulmonary lesions). Therefore, the final sample comprised 44 patients. The mean age of the patients was 58.4 years (range, 22-79 years), and 29 (65.9%) were female. The mean interval between lung biopsy and HRCT was 63 days (range, 1-919 days), that interval being > 180 days in only four patients.

There was moderate to substantial interobserver agreement on HRCT diagnostic categories among all four chest radiologists (κ = 0.61), who agreed in 28 (63.6%) of the 44 cases. Among the 16 remaining cases, there was agreement among three of the four radiologists in 20.5% (n = 9) and between two of the four in 15.9% (n = 7). Considering the binary scores for a typical UIP HRCT pattern versus a probable UIP HRCT pattern and a HRCT pattern indeterminate for UIP versus HRCT features most consistent with a non-IPF diagnosis, we found that there was substantial interobserver agreement among the radiologists (κ = 0.79) in 86.4% of cases. When the radiology resident was included in the analysis, making a total of five raters, there was moderate interobserver agreement regarding the diagnostic categories (κ = 0.59) and substantial interobserver agreement regarding the binary scores (κ = 0.77). The relative frequencies of the typical UIP HRCT pattern, probable UIP HRCT pattern, and HRCT pattern indeterminate for UIP were 4.5-15.9%, 9.1-20.5%, and 11.4-20.5%, respectively.

**Figure 1:**
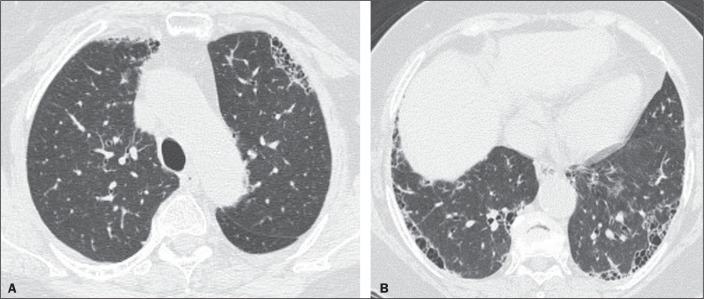
Example of a typical UIP HRCT pattern. A 74-year-old female patient in whom UIP was confirmed by biopsy and who received a final multidisciplinary diagnosis of fibrosing ILD probably secondary to rheumatoid arthritis. All raters agreed regarding the presence of honeycombing and the craniocaudal distribution on HRCT.

**Figure 2:**
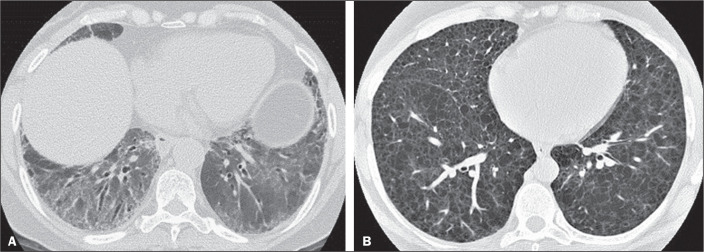
Examples of predominant HRCT imaging patterns. A: A 68-year-old patient with fibrosing ILD that was probably drug-related (according to medical records). There was disagreement among raters regarding the predominant imaging pattern on HRCT, 60% indicating ground-glass opacity, 20% indicating bronchiectasis/bronchiolectasis, and 20% indicating reticulation; 80% of the raters classified these findings as HRCT features most consistent with a non-IPF diagnosis, and 20% classified them as the probable UIP HRCT pattern. B: A 40-year-old patient with biopsy-proven lymphangioleiomyomatosis. All raters agreed on the predominant HRCT imaging pattern (cysts) and diagnostic category.

**Figure 3:**
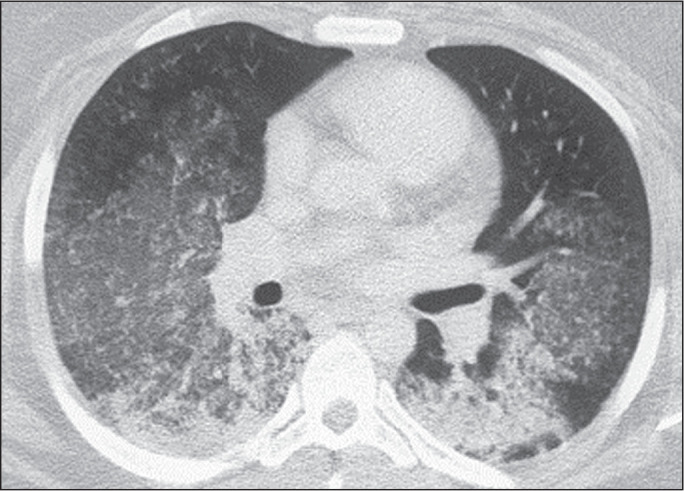
Example of the most prevalent diagnostic category. A 23-year-old female patient diagnosed with alveolar hemorrhage during the investigation of vasculitis, which presented as areas of ground-glass attenuation with central or peribronchovascular consolidation on HRCT. All raters agreed on the diagnostic category of HRCT features most consistent with a non-IPF diagnosis. Four of the five raters included alveolar hemorrhage as one of the diagnostic hypotheses.

For the presence of honeycombing (Figure 1), there was (borderline) almost perfect interobserver agreement among the chest radiologists (κ = 0.81) and substantial interobserver agreement among all raters (κ = 0.69). The κ value found for the identification of the predominant imaging finding was 0.67 among the radiologists and 0.63 among all raters (Figure 2). The most common HRCT diagnostic category among the raters was HRCT features most consistent with a non-UIP diagnosis, which was indicated in 54.5-63.6% of the cases (Figure 3).

For the 44 HRCT scans assessed, 414 diagnostic hypotheses were evaluated, from one to three (as determined in the methodology and collection form) for each patient. The diagnostic hypotheses most frequently considered were nonspecific interstitial pneumonia (92 citations, 22.2%); IPF/UIP (75 citations, 18.1%); hypersensitivity pneumonitis (44 citations, 10.6%); desquamative interstitial pneumonia (20 citations, 4.8%); cryptogenic organizing pneumonia (18 citations, 4.3%); and pulmonary sarcoidosis (16 citations, 3.8%). Other, less frequently cited, hypotheses were lymphocytic interstitial pneumonitis, lymphangioleiomyomatosis, interstitial pneumonia related to collagen diseases, eosinophilic pneumonia, acute interstitial pneumonia, respiratory bronchiolitis-ILD, combined pulmonary fibrosis and emphysema, pulmonary edema, diffuse alveolar damage, alveolar hemorrhage, alveolar proteinosis, infectious diseases, neoplasia (lymphoma), vasculitis, Langerhans cell histiocytosis, drug-induced pulmonary fibrosis, pulmonary amyloidosis, and pneumoconiosis. The list of final diagnoses obtained after histopathological analysis or by multidisciplinary consensus are shown in Table 2.

**Table 2:** Final diagnoses of the 44 patients evaluated.

Final diagnosis	Diagnosis based on histopathology/ multidisciplinary consensus*
UIP (n = 7)	6/1
Nonspecific interstitial pneumonia (n = 10)	9/1
Chronic hypersensitivity pneumonia (n = 9)	5/4
Lymphocytic interstitial pneumonia (n = 3)	2/1
Desquamative interstitial pneumonia (n = 1)	1/-
Respiratory bronchiolitis-interstitial lung disease (n = 4)	4/-
Lymphangioleiomyomatosis (n = 3)	3/-
Diffuse alveolar damage (n = 1)	1/-
Lymphoma (n = 1)	1/-
Cryptogenic organizing pneumonia (n = 1)	1/-
Pneumoconiosis (n = 1)	1/-
Alveolar hemorrhage and vasculitis (n = 1)	1/-
Interstitial pneumonia of inconclusive etiology (n = 2)	2/-
Total	44/7

* When the histopathology was inconclusive.

There was agreement on at least one diagnostic hypothesis among the radiologists in 19 (43.2%) of the 44 cases and among all raters in 16 (36.4%). In addition, there was agreement regarding the most likely diagnosis among the radiologists in 13 cases (29.5%) and among all raters in 12 (27.3%).

For the level of confidence in the most likely diagnostic hypothesis based on the HRCT findings alone, there was slight to fair interobserver agreement (κ = 0.19-0.21). A summary of the results is shown in Table 3.

**Table 3:** Summary of the results obtained for interobserver agreement.

Interobserver agreement variable	Raters: strength of agreement	K		Cases		*P*
		Mean	(range)	n	(%)	
IPF/UIP HRCT diagnostic categories	Chest radiologists: moderate/substantial	0.61	(0.53-0.69)	28	(63.6)	< 0.05
	All: moderate	0.59	(0.53-0.65)	25	(56.8)	< 0.05
HRCT binary score	Chest radiologists: substantial	0.79	(0.67-0.91)	38	(86.4)	< 0.05
	All: substantial	0.77	(0.67-0.86)	36	(81.8)	< 0.05
Presence of honeycombing	Chest radiologists: almost perfect	0.81	(0.68-0.93)			< 0.05
	All: substantial	0.69	(0.60-0.79)			< 0.05
Predominant imaging finding	Chest radiologists: substantial	0.67	(0.60-0.74)			< 0.05
	All: substantial	0.63	(0.58-0.68)			< 0.05
Level of confidence in the most likely diagnostic hypothesis	Chest radiologists: fair	0.21	(0.10-0.31)			< 0.05
	All: none/slight	0.19	(0.11-0.27)			< 0.05
At least one of the three diagnostic hypotheses	—	—	—	16	(36.4)	—
The most likely diagnostic hypothesis	—	—	—	12	(27.3)	—

## DISCUSSION

In the present study, there was moderate to substantial interobserver agreement among raters with different experience levels regarding the diagnostic classification of IPF/UIP based on HRCT findings (κ = 0.59-0.61). As mentioned previously, various studies have evaluated the diagnostic accuracy of HRCT in the context of diffuse lung diseases. Specifically considering the diagnostic classifications of the IPF/UIP, a multicenter study published in 2015^(11)^ stands out for its evaluation of interobserver agreement among 112 raters (including 96 chest radiologists) on 150 consecutive chest HRCT scans of patients with fibrotic lung disease, using the three IPF/UIP diagnostic categories published in the 2011 American Thoracic Society/European Respiratory Society/Japanese Respiratory Society/Latin American Thoracic Association guidelines: UIP; possible UIP; and inconsistent with UIP. The authors of that study found moderate interobserver agreement, with κ values ranging from 0.48 for general radiologists to 0.52 for chest radiologists with 10-20 years of experience.

In another multicenter study, published in 2008, Thomeer et al.^(12)^ evaluated the accuracy of the multidisciplinary diagnosis of 179 patients with IPF (82 undergoing lung biopsy), as well as evaluating interobserver agreement among three radiologists and two pathologists within the current IPF/UIP diagnostic criteria^(13)^. When the HRCT pattern was categorized as improbable, probable, or highly suggestive of UIP, the biopsy was consistent with UIP in 67.5%, 84.5%, and 91.7% of the cases, respectively. Fair interobserver agreement was observed among the three radiologists (κ = 0.40) and between the two pathologists (κ = 0.30). The authors suggested that the high prevalence of UIP in the sample was the cause not only of the low level of interobserver agreement but also of the high number of IPF/UIP cases with an atypical presentation on imaging.

Our results are quite similar to those of Widell et al.^(14)^, who found a κ value of 0.62 for agreement between two raters when the 2018 Fleischner Society criteria were applied (not statistically different from that obtained when the 2011 Fleischner Society criteria were applied). For the presence of honeycombing, those authors found a κ value of 0.81, which is exactly the same as in our study. However, their sample included normal examinations, which could have increased the level of agreement among the raters. In another study that analyzed interobserver agreement based on the most recent (2018) Fleischner Society criteria, the authors found only moderate agreement (κ = 0.50) among six ILD experts, although only one of them was a chest radiologist, the five other raters being pulmonologists^(15)^.

In the present study, there was substantial interobserver agreement (κ = 0.77-0.79) regarding the binary HRCT scores. The evaluation of those scores is extremely valid and justified by its impact on practice. The identification of patients with a typical clinical presentation and a typical or probable UIP HRCT pattern may preclude the need to perform surgical lung biopsy for the definitive diagnosis of IPF^(5)^. The probability of an alternative diagnosis in this group of patients is generally very small and therefore does not justify the risks associated with biopsy^(5,16,17)^. In addition, patients with a typical UIP HRCT pattern show a clinical progression similar to that of those with a probable UIP HRCT pattern, when treated with the same antifibrotic^(18)^. Furthermore, some studies have shown prognostic differences between patients with IPF in whom the HRCT presentation is typical and those in whom it is atypical, the prognosis being better for the latter group. The presentations considered atypical are grouped in the binary score and include the HRCT pattern indeterminate for UIP and HRCT features most consistent with a non-IPF diagnosis^(19)^.

Walsh et al.^(11)^ also analyzed interobserver agreement regarding the binary score for the 2011 American Thoracic Society/European Respiratory Society/Japanese Respiratory Society/Latin American Thoracic Association categories UIP/possible UIP versus inconsistent with UIP, finding moderate interobserver agreement, with κ values ranging from 0.39, for chest radiology fellows, to 0.45, for experienced chest radiologists. Nonetheless, in regions where an interstitial disease other than IPF (e.g., fibrotic hypersensitivity pneumonia) is highly prevalent, lung biopsy should be considered, after cautious risk analysis, if HRCT shows a probable UIP pattern in a patient without known environmental exposure^(20)^. Knowledge of the pretest probability is central to clinical decision-making based on the HRCT pattern. Brownell et al.^(21)^ showed high disparity in the positive predictive values for the probable UIP HRCT pattern between two cohorts in which the prevalence of histological UIP was low and high (62.5 and 94.4%, respectively).

In the present study, it was not our aim to analyze different levels of agreement between experienced and inexperienced raters. However, the greater agreement among all raters regarding the binary scores (in relation to the agreement regarding the four categories separately) may indicate that radiologist experience would have less weight in determining a significant change in practice. That can be beneficial for patient management at non-specialized centers, because less significant disagreements involving less experienced professionals are unlikely to change the practice substantially. However, there is a need for additional studies including a greater number of inexperienced raters, in order to provide further clarification.

For the presence of honeycombing, the interobserver agreement in the present study was substantial to almost perfect (κ = 0.69-0.81), considerably better than the moderate agreement (κ = 0.45-0.59) reported in previous studies^(11,22,23)^. The fact that the prevalence of IPF was lower in our sample than in those of other studies may have contributed to the higher κ values. Another factor to be considered is that the proportion of raters specializing in chest radiology was greater in the present study.

Comparisons between studies analyzing interobserver agreement using the κ statistic can be limited by the heterogeneity between samples and between statistical calculations. The κ statistic is calculated to evaluate the overall agreement between raters, excluding agreement that is attributable to chance. Therefore, the calculated κ statistic is difficult to compare between studies that grouped the variables and those that did not, and even more so between studies employing the old (three-variable) classification and the new (four-variable) classification. In the latter situation, raters might disagree more by chance than because of clinically relevant differences. The κ statistic is influenced by the prevalence of the disease in question, the number of raters, and the number of score categories. The allocation of intermediate scores among raters can influence the final κ value when more than two categories are being analyzed^(24)^. For example, in our study, disagreements between certain categories were considered less relevant, such as those between a typical UIP HRCT pattern and a probable UIP HRCT pattern and those between a HRCT pattern indeterminate for UIP and HRCT features most consistent with a non-IPF diagnosis.

In the present study, there was significant disagreement regarding the level of confidence in the most likely HRCT diagnosis and agreement regarding at least one of the three diagnostic hypotheses in less than half of the cases. In previous studies, the availability of data on interobserver agreement among HRCT raters and diagnostic accuracy vary according to the study design, rater experience, number of patients, and profile of the patient sample, the κ values for interobserver agreement ranging from 0.36 to 0.60. Previous studies have demonstrated that the typical imaging presentation of IPF/UIP has an accuracy of approximately 90% for the diagnosis. In other scenarios, including an atypical presentation, unavailability of clinical data, and radiologist inexperience, the diagnostic accuracy is significantly lower^(12,22,25)^.

The present study has several limitations, primarily the small number of patients. The lower levels of agreement among diagnostic hypotheses can be alarming when evaluated in isolation. The overlapping of imaging presentations among ILD and the unavailability of some clinical data should be considered factors that influenced the results. In addition, our study sample was composed only of patients undergoing surgical lung biopsy, and the prevalence of atypical imaging presentations was therefore higher. Therefore, greater difficulty in determining a specific diagnosis is expected, given that surgical lung biopsy is most commonly indicated in cases in which the clinical and radiological findings are inconclusive or conflicting. That sample selection strategy might also explain the fact that HRCT features most consistent with a non-IPF diagnosis was the most prevalent HRCT pattern. Furthermore, this subgroup of patients (patients with ILD undergoing surgical lung biopsy) likely corresponds to the most challenging cases and therefore the greatest potential to improve the diagnostic accuracy, which increases the relevance of the results obtained. Therefore, despite the aforementioned limitations, the results of this study should be valued, especially when HRCT plays a central role in diagnostic and therapeutic decisions regarding patients with ILD.

## CONCLUSION

There was moderate to substantial interobserver agreement regarding the HRCT IPF/UIP diagnostic categories and substantial interobserver agreement regarding the binary HRCT scores among radiologists with different levels of experience. A major limitation of the HRCT method was demonstrated in determining specific diagnostic hypotheses, attributable, at least in part, to the large number of cases with atypical imaging presentations and the unavailability of some clinical and laboratory data. There is a need for multicenter studies, evaluating larger patient samples, in order to evaluate this topic further.
